# Association between serum PCSK9 and coronary heart disease in patients with type 2 diabetes mellitus

**DOI:** 10.1186/s13098-023-01238-z

**Published:** 2023-12-20

**Authors:** Juan Huang, Jun-Xu Gu, Kun Wang, Ai-Min Zhang, Ting-Ting Hong, Shan-Shan Li, Xiao-Qin Yao, Ming Yang, Yue Yin, Na Zhang, Ming Su, Jia-Jia Hu, Xue-Zhi Zhang, Mei Jia

**Affiliations:** 1 Department of traditional Chinese medicine, Peking University International Hospital, No. 1 Shengmingyuan Road, Zhongguancun Life Science Park, Changping District, Beijing, 102206 P.R. China; 2 Department of Clinical Laboratory, Peking University People’s Hospital, No. 11 Xizhimen South Street, Beijing, 100044 P.R. China; 3 Department of Clinical Laboratory, Xiangya Hospital, Central South University, Changsha, P.R. China; 4 Department of Clinical Laboratory, Peking University International Hospital, Beijing, P.R. China

**Keywords:** PCSK9, T2DM, Coronary heart disease, Cardiovascular events

## Abstract

**Background and aims:**

Proprotein convertase subtilisin/kexin type 9 (PCSK9) is considered a new biomarker for atherosclerosis, but its ability to predict cardiovascular outcomes has been controversial. This study aimed to address the lack of data on PCSK9, coronary heart disease (CHD) severity, and major cardiovascular events (MACEs) in patients with type 2 diabetes mellitus (T2DM).

**Methods:**

A total of 2984 T2DM patients underwent selective coronary angiography, and their serum PCSK9 levels were measured using enzyme-linked immunosorbent assay. Correlation and logistic regression analyses were performed to investigate the association between PCSK9 expression and CHD severity. This study used Cox regression analysis to assess the association between circulating PCSK9 levels and the risk of MACEs.

**Results:**

Circulating PCSK9 levels were significantly higher in the CHD group than in the non-CHD group [554.62 (265.11) ng/mL vs. 496.86 (129.05) ng/mL, *p* < 0.001]. Circulating PCSK9 levels positively correlated with CHD severity (diseased vessels: r = 0.35, *p* < 0.001; Gensini score: r = 0.46, *p* < 0.001). Elevated PCSK9 levels are an independent risk factor for CHD risk and severity (CHD group vs. non–CHD group: OR = 2.829, 95% CI: 1.771–4.520, *p* < 0.001; three vessel disease group vs. one vessel disease group: OR = 4.800, 95% CI: 2.387–9.652, *p* < 0.001; high GS group vs. low GS group: OR = 5.534, 95% CI: 2.733–11.208, *p* < 0.001). Through a six-year follow-up and multivariate Cox regression analysis, elevated circulating PCSK9 levels were found to be independently associated with MACEs in all participants (HR: 3.416, 5% CI: 2.485–4.697, *p* < 0.001; adjusted HR: 2.780, 95% CI: 1.930–4.004, *p* < 0.001).

**Conclusions:**

Serum PCSK9 levels were positively correlated with multi-vessel CHD and Gensini score. Elevated circulating PCSK9 levels are an independent risk factor for CHD and increased incidence of MACEs in T2DM.

**Supplementary Information:**

The online version contains supplementary material available at 10.1186/s13098-023-01238-z.

## Introduction

Coronary heart disease (CHD) is a prevalent cardiovascular disease and is the leading cause of disability and death in humans [1–3]. Over the past half a century, several studies have shown that diabetes, hypertension, dyslipidemia, and smoking are possible risk factors for cardiovascular disease that can be used for the early assessment of the risk for cardiovascular disease [4, 5]. Type 2 diabetes mellitus (T2DM) has a variety of causes that lead to insufficient insulin secretion or the inability of the body to effectively use insulin, resulting in a sustained increase in blood sugar levels and impaired carbohydrate, lipid, and protein metabolism. It mainly occurs in adults with obesity, and the number of females with T2DM is greater than that of males with T2DM [6, 7]. T2DM patients are prone to lipid metabolism disorders, thereby having increased risk of cardiovascular diseases [8, 9].

The proprotein convertase subtilisin/kexin type 9 (PCSK9) is a member of the proprotein convertase enzyme family [10, 11]. PCSK9 is a serine protease mainly derived from the liver and intestines; however, according to animal studies, the primary source of circulating PCSK9 is the liver [12, 13]. PCSK9 regulates the degradation of low-density lipoprotein receptors (LDLR) on the surface of liver cells via the endosome/lysosome pathway, revealing a new concern in low-density cholesterol homeostasis. In addition to this classical pathway, recent research has also found that PCSK9 plays other roles in the development of atherosclerosis via non-classical mechanisms, such as inflammation, apoptosis and immune pathways [11, 14, 15].

In recent years, many studies have shown that PCSK9 can promote the occurrence and development of vascular atherosclerosis by promoting an inflammatory response and endothelial dysfunction and inhibiting platelet activation [16, 17]. Several large-scale clinical trials have found that human circulating PCSK9 monoclonal antibodies can significantly reduce serum low-density lipoprotein cholesterol (LDL-C) levels and demonstrate outstanding potential for reducing future cardiovascular events. Most importantly, circulating PCSK9 has been recognized as an adverse cardiovascular risk factor in patients with CAD [18, 19]. Owing to the close relationship between lipid and glucose metabolism, an increasing number of studies have found a close relationship between PCSK9 and diabetes [20, 21]. However, the pathogenicity of PCSK9 in T2DM patients and T2DM patients with CHD has not been completely established.

Therefore, this study aims to investigated serum PCSK9 levels and severity of CHD in stable CHD patients with T2DM, as well as the risk of long-term cardiovascular events.

## Methods

### Study population

**Fig. 1:**
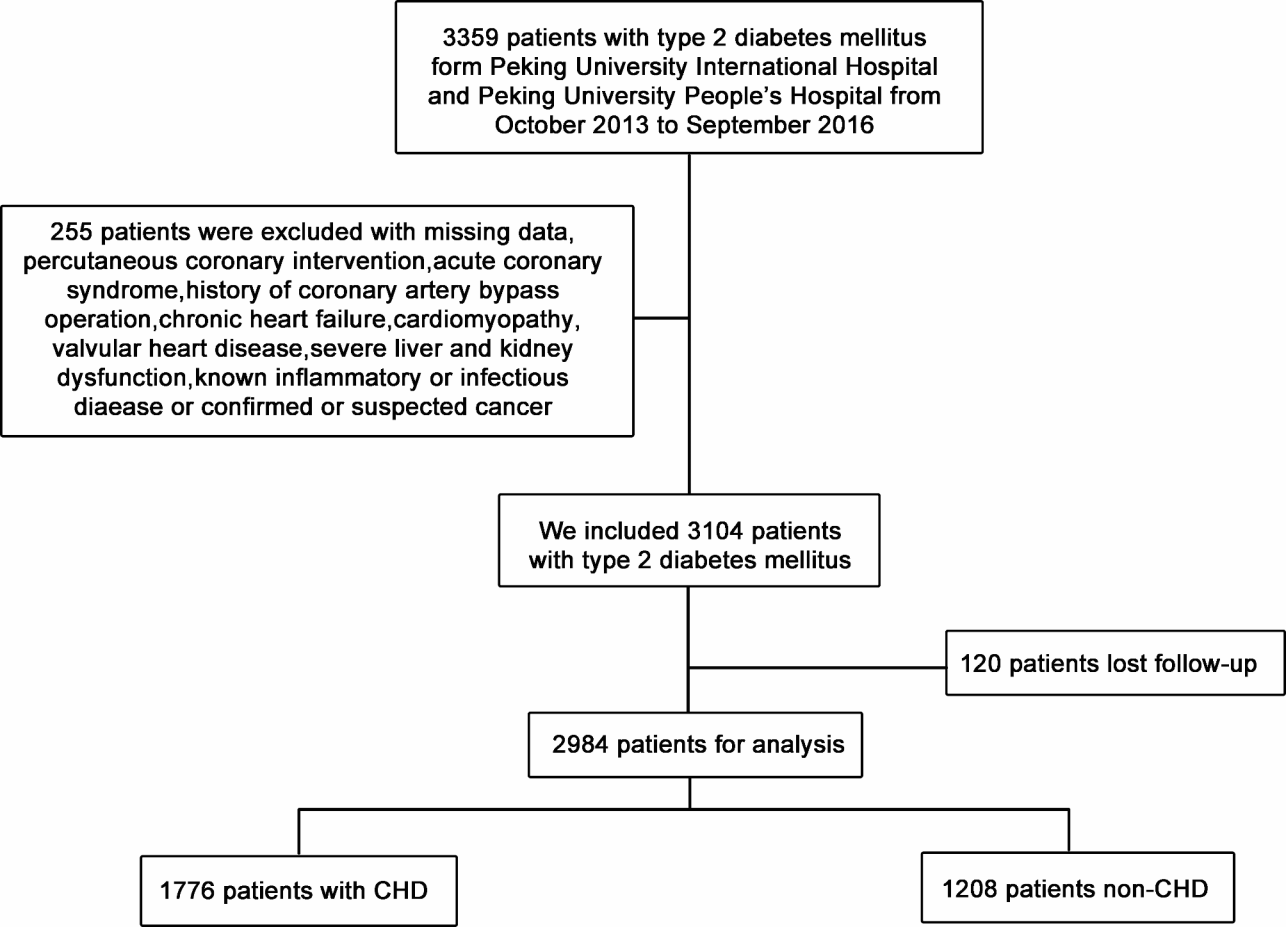
The flow chart of the patients’ selection process

This is a cohort study. This study enrolled 2984 T2DM patients (1732 males, 1252 females) from Peking University International Hospital and Peking University People’s Hospital between October 2013 and September 2016. The participants included 1776 patients (1046 males, 730 females) with CHD and 1208 (686 males, 522 females) without CHD. The follow-up procedures were performed every six months by experienced nurses or doctors through telephone or face-to-face interviews. This is illustrated in the flowchart (Fig. 1). Major cardiovascular events (MACEs) include cardiovascular mortality, non-fatal myocardial infarction (MI), non-fatal stroke, heart failure, and hospitalization for unstable angina. The longest follow-up time among all patients was six years.

All patients diagnosed with T2DM were selected according to the following criteria set by the American Diabetes Association: (1) currently receiving treatment of oral hypoglycemic medicine or insulin, (2) self-reporting to the clinician that they have a history of type 2 diabetes, (3) repeated fasting plasma glucose (FPG) greater than 7.0 mmol/L, or (4) glycated hemoglobin A1c (HbA1c) ≥ 6.5%.

The diagnostic criteria for patients with CHD were based on the results of coronary angiography (CAG) performed by Peking University International Hospital or Peking University People’s Hospital, which is defined as at least one major coronary artery occlusion or stenosis exceeding 50%, and the severity of CHD was evaluated using the Gensini score (GS).

Exclusion criteria included: (1) a history of coronary artery bypass surgery; (2) percutaneous coronary intervention within three months; (3) acute coronary syndrome within six months; (4) cardiomyopathy, valvular heart disease, or chronic heart failure; (5) pulmonary heart disease; (6) severe liver and kidney dysfunction; or (7) any known inflammatory or infectious disease, or confirmed, or suspected cancer.

This study complied with the Declaration of Helsinki and was approved by the Peking University People’s Hospital Research Ethics Committee (No.2018PHB155-01). Written informed consent was obtained from all the enrolled patients.

### Bioinformatics analysis of PCSK9

Protein and protein network analysis STRING (https://string-preview.org/) was used to construct and analyze protein and protein networks based on PCSK9.Enriched Gene Ontology and Kyoto Encyclopedia of Genes and Genomes pathways were evaluated using gene set enrichment analysis.

### Conventional clinical and laboratory indicator tests

Blood samples were collected in the morning after fasting for 12 h. Serum samples were prepared by centrifugation at 3500 rpm for 10 min at 10–15 °C and were stored at − 80 °C.

The circulating levels of PCSK9 were measured using the ELISA kit (Duduo Biotechnology Co., Ltd. China), according to the manufacturer’s instruction. Fasting blood glucose (FBG), hypersensitive C-reactive protein (hs-CRP), homocysteine (HCY), and serum lipid profiles, including triglycerides (TG), total cholesterol (TC), LDL-C, and high-density lipoprotein cholesterol (HDL-C), were analyzed using the Beckman AU5832 analyzer (Beckman Coulter Inc., USA). Lipoprotein (a) [Lp(a)] was measured using the latex enhanced immunoturbidimetry Lp(a) kit (Roche Inc., Germany). Apolipoprotein A-1 (apoA1) and B (apoB) were measured using immunoturbidimetry (Daiichi Pure Chemicals Co., Ltd., Tokyo). Small dense low-density lipoprotein cholesterol (sdLDL-C) was detected using sdLDL-C direct quantitative analysis kits (Denka Seiken Co., Ltd. Japan). Hemoglobin A1c (HbA_1c_) levels were determined using high-performance liquid chromatography (Trinity Biotech Inc., USA).

### Statistical analyses

The one-sample Kolmogorov Smirnov test was used to analyze the distribution of all quantitative variables. Normally distributed data are presented as mean ± standard deviation, and the Student’s t-test and the analysis of variance were used to compare the differences between groups. Continuous data with non-normal distribution are reported as medians (inter-quartile ranges), and differences between various groups were compared using the Mann–Whitney U test and Kruskal–Wallis test. Categorical data were presented as percentages (%) and compared using the chi-square test. Spearman’s correlation analysis was used to calculate the correlation coefficient between the circulating PCSK9 levels and CHD severity. The relationship between PCSK9 and CHD severity was analyzed using univariate and multivariate logistic analyses. The Kaplan–Meier method was used to estimate the survival rate of MACEs in the groups. Hazard ratios (HR) and 95% confidence intervals (CI) for the relationship between circulating PCSK9 levels and the incidence of MACEs were generated using univariate and multivariate Cox regression analyses. *p* < 0.05 was considered statistically significant. SPSS 22.0 for Windows (SPSS Inc., USA) and GraphPad Prism 7 (GraphPad Software Inc., USA) were used for statistical analyses.

## Results

### Baseline characteristics of all patients

We initially analyzed the baseline characteristics of all participants. Table 1 presents the demographic, anthropometric, and biochemical characteristics of the study population. There were no significant differences between the CHD and non-CHD groups in terms of sex (male), age, body mass index (BMI), percentage of hypertension, alcohol consumption, smoking, family history of diabetes mellitus (MD), FBG levels, HbA_1c_, TG, hs-CRP and HCY. In contrast, the CHD group showed a higher percentage of a family history of CHD than the non-CHD group. In addition, a statistically significant increase in ApoB, TC, LDL-C, Lp(a), sdLDL-C, and PCSK9 levels [554.62 (265.11) ng/ml vs. 496.86 (129.05) ng/ml, *p* < 0.001] was observed, whereas ApoA1 and HDL-C levels decreased in the CHD group.

**Table 1:** Baseline characteristics in type 2 diabetic patients

	Total	CHD group	Non-CHD group	*p* value
**Clinical characteristics**				
N (%)	2984	1776 (59.52%)	1208 (40.48%)	–
Age (years)	57.48 ± 9.93	57.81 ± 9.74	56.99 ± 10.19	0.222
Male (%)	1732 (58.04%)	1046 (58.90%)	686 (56.78%)	0.252
BMI (kg/m^2^)	25.23 ± 3.07	25.06 ± 2.90	25.48 ± 3.31	0.131
Hypertension (%)	2084 (69.53%)	1252 (70.50%)	832 (68.87%)	0.344
Smoking (%)	838 (28.08%)	481 (27.08%)	357 (29.55%)	0.141
Alcohol consumption (%)	887 (29.73%)	541 (30.46%)	346 (28.64%)	0.286
Family history of CHD (%)	851 (28.52%)	531 (29.90%)	320 (26.49%)	0.043
Family history of MD (%)	1135 (38.04%)	655 (36.88%)	480 (39.73%)	0.115
**Laboratory variables**				
FBG (mmol/L)	7.69 ± 1.16	7.73 ± 1.21	7.64 ± 1.08	0.219
HbA1c (%)	7.52 ± 1.10	7.55 ± 1.15	7.48 ± 1.02	0.367
ApoB (mg/dL)	85.10 (26.85)	87.00 (36.60)	84.16 (20.16)	0.004
ApoA1 (mg/dL)	144.1 (37.55)	142.50 (41.50)	145.65 (31.58)	0.014
Total cholesterol (mmol/L)	4.44 (1.34)	4.46 (1.72)	4.41 (0.98)	0.009
Triglycerides (mmol/L)	1.32 (0.88)	1.32 (0.82)	1.32 (0.94)	0.395
HDL-C (mmol/L)	1.14 (0.35)	1.13 (0.30)	1.15 (0.39)	0.005
LDL-C (mmol/L)	2.73 (0.94)	2.79 (1.37)	2.71 (0.67)	0.011
Lp(a) (nmol/L)	39.24 (39.77)	40.51 (48.21)	36.99 (33.45)	0.001
hs-CRP (mg/L)	1.38 (1.69)	1.57 (1.80)	1.23 (1.56)	0.386
HCY (umol/L)	11.65 (7.69)	11.77 (8.51)	11.45 (6.60)	0.091
sdLDL-C (mmol/L)	0.74 (0.37)	0.76 (0.47)	0.71 (0.31)	0.005
PCSK9 (ng/mL)	521.98 (209.78)	554.62 (265.11)	496.86 (129.05)	< 0.001

Data are reported as means ± SD or n(%), median (interquartile ranges). SD: Standard deviation

BMI: body mass index; FPG: fasting plasma glucose; HbA1c: Hemoglobin A1c; apoB: apolipoprotein B; apoA1: apolipoprotein A1; HDL-C: high density lipoprotein cholesterol; LDL-C: low density lipoprotein cholesterol; Lp(a): lipoprotein (a); Hs-CRP: hypersensitive C-reactive protein; HCY: homocysteine; sdLDL-C: small dense low-density lipoprotein cholesterol; PCSK9: proprotein convertase enzyme subtilisin/kexin type 9

Statistical analysis was performed with the Student’s t test or Mann-Whitney U test and with Chi-square test for categorical variables

Next, we divided all the patients into four subgroups based on the quartile of PCSK9 levels (Table 2). ApoA1 and HDL-C levels were higher in the lowest quartile than in the highest quartile of PCSK9 levels (all *p* < 0.05). Furthermore, the levels of Lp(a), TC, LDL-C, ApoB, HCY, and sdLDL-C were significantly higher in participants in the highest quartile of PCSK9 levels than in those in the lowest quartile of PCSK9 levels (all *p* < 0.05). In addition, there were no significant differences in age, sex (male), BMI, percentage of hypertension, smoking, alcohol consumption, family history of CHD, family history of DM, FBG levels, HbA_1c_, TG and hs-CRP between the two groups (all *p* > 0.05).

**Table 2:** Baseline characteristics of patients with type 2 diabetes at different circulating PCSK9 levels

Variables	PCSK9 concentration (ng/mL)				*p* value
	Q1: < 432.98	Q2: 432.98–521.98	Q3: 521.98–621.24	Q4: > 621.24	
**Clinical characteristics**					
N (%)	748 (25.07%)	744 (24.93%)	746 (25.00%)	746 (25.00%)	–
Age (years)	57.07 ± 9.19	57.59 ± 9.94	56.94 ± 10.03	58.31 ± 10.33	0.440
Male (%)	437 (58.42%)	419 (56.32%)	451 (60.46%)	425 (56.97%)	0.378
BMI (kg/m^2^)	24.91 ± 2.82	25.55 ± 3.54	25.47 ± 2.98	24.97 ± 2.85	0.203
Hypertension (%)	501 (66.98%)	532 (71.51%)	541 (72.52%)	510 (68.36%)	0.066
Smoking (%)	220(29.41%)	193(25.94%)	215(28.82%)	210(28.15%)	0.467
Alcohol consumption (%)	232(31.02%)	236(31.72%)	216(28.95%)	203(27.21%)	0.214
Family history of CHD (%)	194(25.94%)	201(27.02%)	220(29.49%)	236(31.64%)	0.067
Family history of MD (%)	272(36.36%)	293(39.38%)	280(37.53%)	290(38.87%)	0.623
**Laboratory variables**					
FPG (mmol/L)	7.62 ± 1.10	7.59 ± 1.11	7.80 ± 1.15	7.78 ± 1.21	0.057
HbA1c (%)	7.44 ± 1.06	7.44 ± 1.02	7.58 ± 1.10	7.62 ± 1.19	0.181
ApoB (mg/dL)	83.10 (26.80)	81.56 (23.96)	84.48 (25.47)	92.95 (31.95)^abc^	< 0.001
ApoA1 (mg/dL)	150.70 (33.05)	145.71 (48.11)	142.05 (32.93)^a^	140.50 (32.99)^ab^	0.001
Total cholesterol (mmol/L)	4.34 (1.42)	4.32 (1.29)	4.41 (1.19)	4.64 (1.49)^abc^	< 0.001
Triglycerides (mmol/L)	1.33 (0.82)	1.28 (0.96)	1.31 (0.95)	1.36 (0.82)	0.997
HDL-C (mmol/L)	1.16 (0.41)	1.13 (0.38)	1.12 (0.27)^a^	1.12 (0.35)^a^	0.023
LDL-C (mmol/L)	2.66 (1.00)	2.69 (0.81)	2.73 (0.85)^a^	3.02 (1.17)^abc^	< 0.001
Lp(a) (nmol/L)	30.67 (19.41)	36.54 (26.13)^a^	40.33 (35.36)^a^	76.71 (84.78)^abc^	< 0.001
hs-CRP (mg/L)	1.33 (1.62)	1.51 (1.85)	1.31 (1.79)	1.34 (1.55)	0.948
HCY (umol/L)	11.21 (6.62)	11.40 (7.14)	11.33 (6.62)	13.56 (9.80)^abc^	< 0.001
sdLDL-C (mmol/L)	0.73 (0.43)	0.69 (0.43)	0.75 (0.35)	0.79 ( 0.47)^ab^	0.008
PCSK9 (ng/mL)	379.84 (83.46)	480.04 (47.30)	566.52 (44.78)	765.66 (200.58)	

Data are reported as means ± SD or n(%), median (interquartile ranges). SD: Standard deviation

BMI: body mass index; FPG: fasting plasma glucose; HbA1c: Hemoglobin A1c; apoB: apolipoprotein B; apoA1: apolipoprotein A1; HDL-C: high density lipoprotein cholesterol; LDL-C: low density lipoprotein cholesterol; Lp(a): lipoprotein (a); Hs-CRP: hypersensitive C-reactive protein; HCY: homocysteine; sdLDL-C: small dense low-density lipoprotein cholesterol; PCSK9: proprotein convertase enzyme subtilisin/kexin type 9

Statistical analysis was performed with the ANOVA or Kruskal – Wall test and with Chi-square test for categorical variables

a: Shows that the *p* < 0.05 compared with the Q1 group

b: Shows that the *p* < 0.05 compared with the Q2 group

c: Shows that the *p* < 0.05 compared with the Q3 group

### Association between PCSK9 and the severity of CHD

**Fig. 2:**
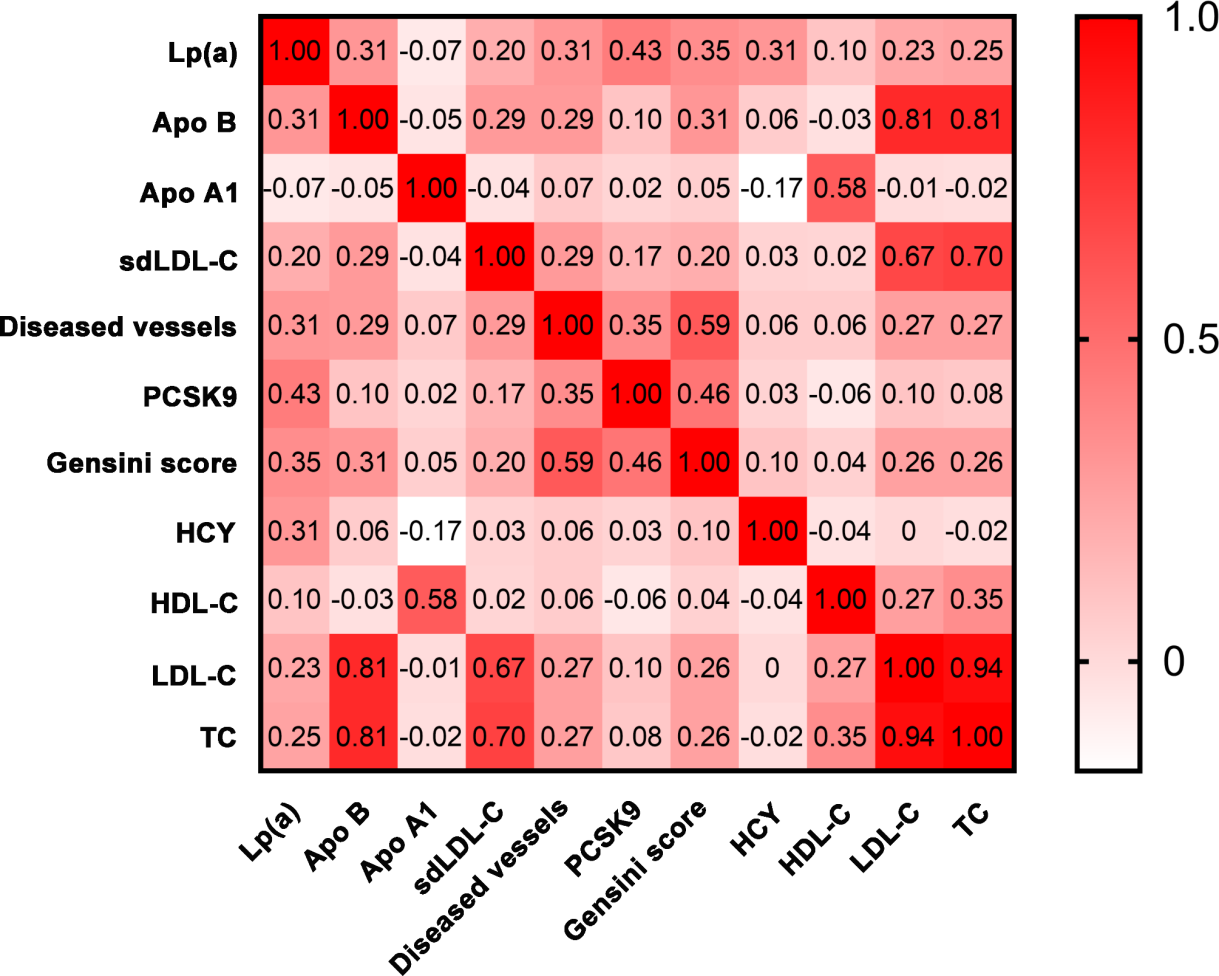
Correlation between PCSK9 and severity of coronary heart disease (diseased vessels: r = 0.35, *p* < 0.001; Gensini score: r = 0.46, *p* < 0.001)

To explore the relationship between PCSK9 and CHD severity, we divided the patients in the CHD group into four subgroups based on the quartile of PCSK9 levels (Supplementary Table 1). We observed that the percentage of patients with three-vessel disease (17.30% vs. 31.01% vs. 50.45% vs. 66.52%, *p* < 0.001) and GS [23 (21) vs. 30 (21) vs. 36 (24) vs. 46 (20), *p* < 0.001] increased in the high PCSK9 group, whereas the percentage of patients with one-vessel disease decreased (*p* < 0.001), indicating that PCSK9 levels may be associated with CHD severity. Furthermore, Spearman’s correlation analysis showed that serum PCSK9 levels were significantly and positively associated with diseased vessels and GS (r = 0.35, *p* < 0.001; r = 0.46, *p* < 0.001, respectively) (Fig. 2).

We also performed a protein–protein interaction analysis on PCSK9 by using STRING and the results were as shown in Supplementary Materials Fig. 1. In addition, GO annotations and KEGG signaling pathway analysis for these related genes and proteins were provided in Supplementary Materials Fig. 2.

### Serum PCSK9 is associated with the risk of CHD in diabetic patients

Based on the CAG examination reports of each patient, diabetic patients with CHD were further divided into one-, two-, and three-vessel disease groups, or low- and high-GS groups, to further evaluate the relationship between PCSK9 and the severity of CHD.

Multivariate binary logistic regression was employed to analyze the relationship between PCSK9 and CHD risk and severity. All participants were divided into subgroups according to PCSK9 level quartiles, and the lowest PCSK9 quartile group was used as a reference to assess the risk and severity of CHD in individuals with different PCSK9 levels.

**Fig. 3:**
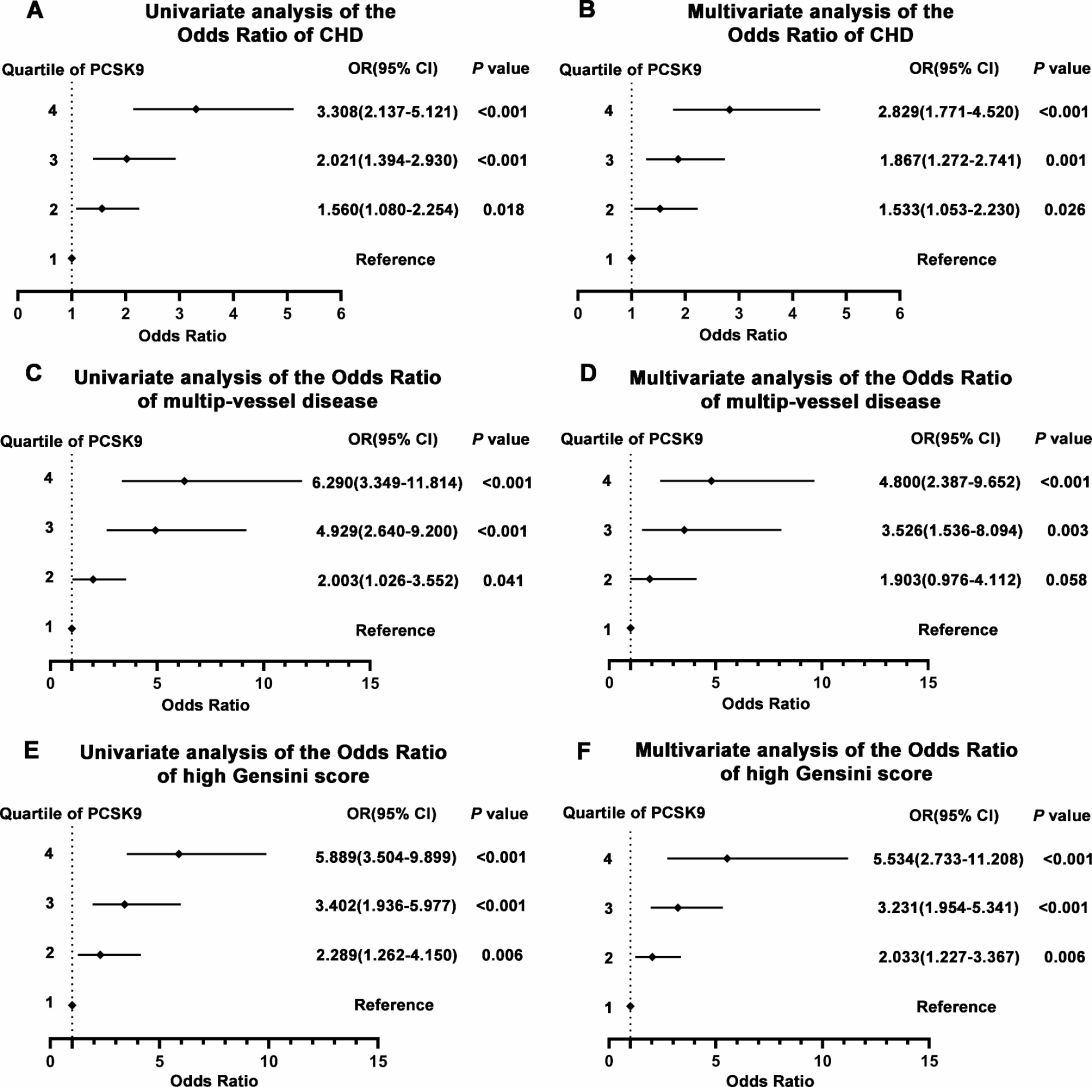
Logistic regression analysis between circulating PCSK9 quartile and CHD, multiple-vessel disease and high Gensini score. (**A**) univariate logistic regression analysis between circulating PCSK9 and CHD; (**B**) multivariate logistic regression analysis between circulating PCSK9 and CHD; (**C**) univariate logistic regression analysis between circulating PCSK9 and multiple-vessel disease; (**D**) multivariate logistic regression analysis between circulating PCSK9 and multiple-vessel disease; (**E**) univariate logistic regression analysis between circulating PCSK9 and Gensini score; (**F**) multivariate logistic regression analysis between circulating PCSK9 and Gensini score

Univariate logistic regression analysis showed that the ORs for CHD risk and severity were positively associated with PCSK9 levels (CHD group vs. non–CHD group: OR = 3.308, 95% CI: 2.137–5.121, *p* < 0.001; three vessel disease group vs. one vessel disease group: OR = 6.290, 95% CI: 3.349–11.814, *p* < 0.001; high GS group vs. low GS group: OR = 5.889, 95% CI: 3.504–9.899, *p* < 0.001) (Fig. 3, A, C, E). Furthermore, the upward trend remained even after adjusting for age, sex, BMI, hypertension, smoking, alcohol consumption, family history of CHD, Family history of DM, FPG, HbA_1c_, ApoB, ApoA1, TC, TG, HDL-C, LDL-C, Lp(a), hs-CRP, HCY and sdLDL-C levels compared with those in the first quartile of PCSK9 (CHD group vs. non–CHD group: OR = 2.829, 95% CI: 1.771–4.520, *p* < 0.001; three vessel disease group vs. one vessel disease group: OR = 4.800, 95% CI: 2.387–9.652, *p* < 0.001; high GS group vs. low GS group: OR = 5.534, 95% CI: 2.733–11.208, *p* < 0.001) (Fig. 3, B, D, F). Taken together, these data indicate that PCSK9 levels are positively correlated with CHD severity.

### PCSK9 and cardiovascular outcomes

**Fig. 4:**
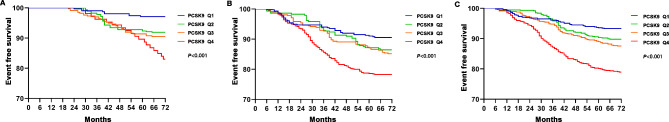
Kaplan-Meier curve based on circulating PCSK9 quartile values among different patients. (**A**) in the non-CHD group; (**B**) in the CHD group; (**C**) in all participants

After six years of follow-up, 373 (12.50%) individuals developed MACEs. In the non-CHD group, 138 individuals were diagnosed with CHD, and 96 (7.95%) of these patients had MACEs. In the CHD group, 277 (15.60%) patients experienced MACEs. Patients with MACEs had presented with clinical features, such as high percentage of family history of CHD, ApoB, TC, sdLDL-C, and PCSK9 [644.66 (310.41) ng/ml vs. 511.01 (169.12) ng/ml, *p* < 0.001] (Table 3). Based on the quartiles of PCSK9 levels, all 2984 individuals were divided into four subgroups to investigate the relationship between the levels of PCSK9 and MACEs risk. As shown in Supplementary Tables 2, 3, 4, 5 and 6, the total incidence of MACEs in the highest PCSK9 quartile group was significantly higher than that in the other three groups, including the non-CHD group (2.90% vs. 8.11% vs. 9.52% vs. 16.98%, *p* < 0.001), the CHD group (9.36% vs. 13.54% vs. 14.88% vs. 21.25%, *p* < 0.001), all the participants (6.68% vs. 10.22% vs. 12.47% vs. 20.64%, *p* < 0.001), male participants (6.76% vs. 11.58% vs. 12.91% vs. 20.68%, *p* < 0.001), and female participants (6.58% vs. 8.58% vs. 11.76% vs. 20.59%, *p* < 0.001). Furthermore, Kaplan–Meier analysis showed that the incidence of MACEs in the highest PCSK9 quartile group was higher than that in the other three groups (Fig. 4C, p < 0.001). The results were similar between the non-CHD and CHD groups (Fig. 4A and B, all *p* < 0.001).

**Table 3:** Baseline characteristics in all subjects with or without MACEs

	Total	MACEs group	Non- MACEs group	*p* value
**Clinical characteristics**				
N (%)	2984	373 (12.50%)	2611 (87.50%)	–
Age (years)	57.48 ± 9.93	58.42 ± 11.37	57.34 ± 9.69	0.265
Male (%)	1732 (58.04%)	225 (60.32%)	1507 (57.72%)	0.340
BMI (kg/m^2^)	25.23 ± 3.07	24.89 ± 2.62	25.27 ± 3.14	0.329
Hypertension (%)	2084 (69.53%)	276 (73.99%)	1808 (69.25%)	0.062
Smoking (%)	838 (28.08%)	108 (28.95%)	730 (27.96%)	0.689
Alcohol consumption (%)	887 (29.73%)	110 (29.49%)	777 (29.75%)	0.916
Family history of CHD (%)	851 (28.52%)	131 (35.12%)	720 (27.58%)	0.003
Family history of MD (%)	1135 (38.04%)	146 (39.14%)	989 (37.88%)	0.638
**Laboratory variables**				
FPG (mmol/L)	7.69 ± 1.16	7.73 ± 1.13	7.68 ± 1.15	0.679
HbA1c (%)	7.52 ± 1.10	7.58 ± 1.13	7.51 ± 1.09	0.492
ApoB (mg/dL)	85.10 (26.85)	90.00 (33.90)	84.52 (25.79)	0.008
ApoA1 (mg/dL)	144.1 (37.55)	144.35 (34.57)	144.10 (37.70)	0.874
Total cholesterol (mmol/L)	4.44 (1.34)	4.59 (1.30)	4.43 (1.33)	0.013
Triglycerides (mmol/L)	1.32 (0.88)	1.37 (0.91)	1.31 (0.87)	0.411
HDL-C (mmol/L)	1.14 (0.35)	1.09 (0.37)	1.14 (0.35)	0.257
LDL-C (mmol/L)	2.73 (0.94)	2.73 (1.16)	2.73 (0.92)	0.161
Lp(a) (nmol/L)	39.24 (39.77)	39.51 (41.25)	39.31 (40.03)	0.527
hs-CRP (mg/L)	1.38 (1.69)	1.32 (1.73)	1.39 (1.66)	0.312
HCY (umol/L)	11.65 (7.69)	11.79 (6.88)	11.62 (7.86)	0.941
sdLDL-C (mmol/L)	0.74 (0.37)	0.80 (0.39)	0.73 (0.37)	0.048
PCSK9 (ng/mL)	521.98 (209.78)	644.66 (310.41)	511.01 (169.12)	< 0.001

Data are reported as means ± SD or n(%), median (interquartile ranges). SD: Standard deviation

BMI: body mass index; FPG: fasting plasma glucose; HbA1c: Hemoglobin A1c; apoB: apolipoprotein B; apoA1: apolipoprotein A1; HDL-C: high density lipoprotein cholesterol; LDL-C: low density lipoprotein cholesterol; Lp(a): lipoprotein (a); Hs-CRP: hypersensitive C-reactive protein; HCY: homocysteine; sdLDL-C : small dense low-density lipoprotein cholesterol

Statistical analysis was performed with the Student’s t test or Mann-Whitney U test and with Chi-square test for categorical variables

**Fig. 5:**
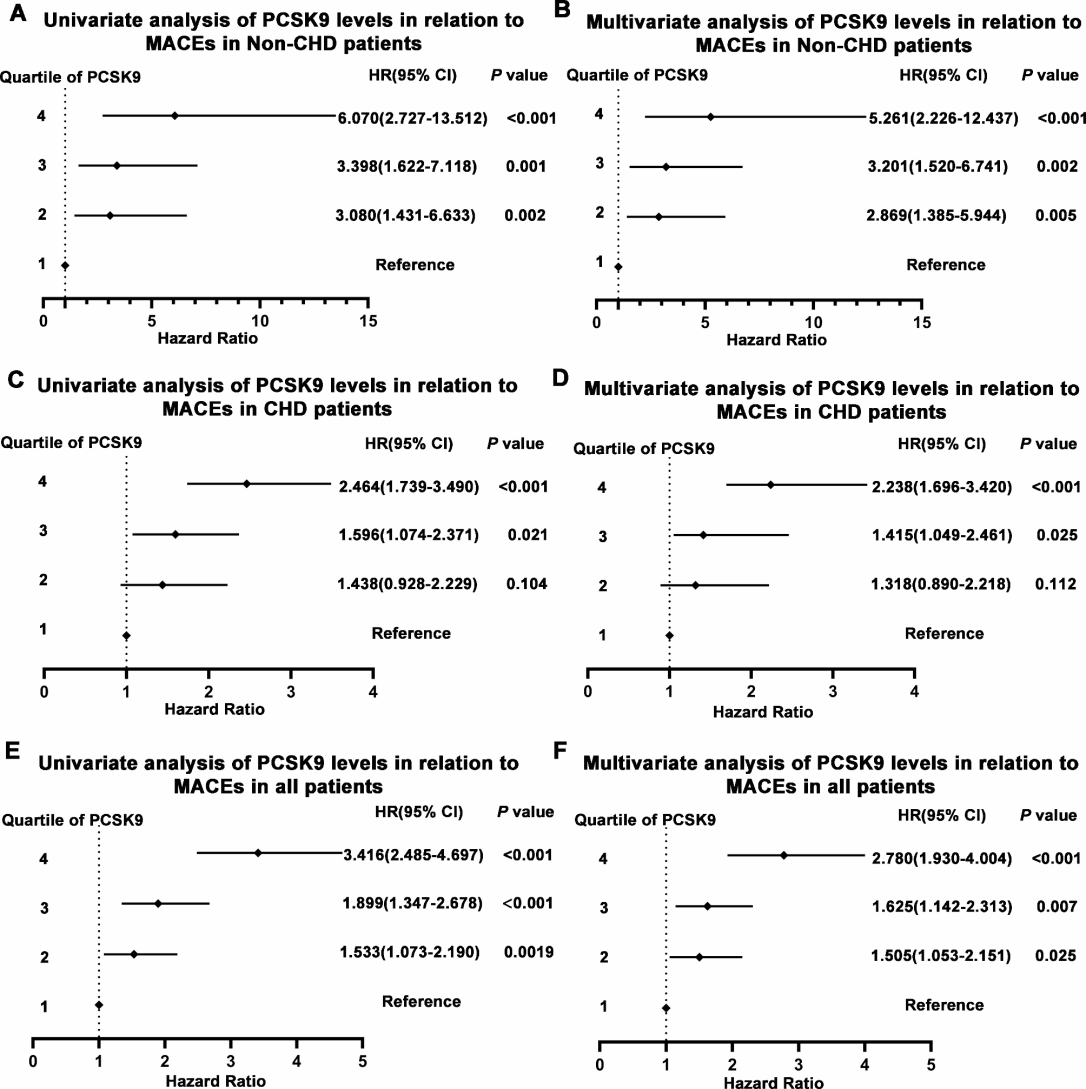
Cox regression analysis of circulating PCSK9 quartile in different patients. (**A**) univariate Cox regression analysis in non-CHD group; (**B**) multivariate Cox regression analysis in non-CHD group; (**C**) univariate Cox regression analysis in CHD group; (**D**) multivariate Cox regression analysis in CHD group; (**E**) univariate Cox regression analysis in all participants; (**F**) multivariate Cox regression analysis in all participants

According to the univariate Cox regression analysis, there was a 6.070-fold higher risk of MACEs in the highest PCSK9 quartile group than that in the lowest (reference group) in the non-CHD group (HR: 6.070, 95% CI: 2.727–13.512, *p* < 0.001) (Fig. 5A). A significant association was observed after adjusting for confounding factors (HR: 5.261, 95% CI: 2.226–12.437, *p* < 0.001) (Fig. 5B). In the CHD group and all the participants, the results were consistent with those of the non-CHD group in both univariate and multivariate Cox regression analyses (CHD: HR: 2.464, 95% CI: 1.739–3.490, *p* < 0.001; adjusted HR: 2.238, 95% CI: 1.696–3.420, *p* < 0.001. All subjects: HR: 3.416, 95% CI: 2.485–4.697, *p* < 0.001; adjusted HR: 2.780, 95% CI: 1.930–4.004, *p* < 0.001) (Fig. 5C–F; Supplementary Table 7). These data, indicate that the PCSK9 level is related to MACEs in all the participants.

## Discussion

In this study, we investigated the association between circulating PCSK9 and the risk and severity of CHD, by evaluating circulating PCSK9 as a predictive biomarker for cardiovascular events in T2DM patients. We found that circulating PCSK9 was positively associated with the risk of CHD in patients with T2DM and was positively associated with the severity of CHD. We also found that higher PCSK9 levels indicated higher rates of cardiovascular events.

To the best of our knowledge, this is the first large-scale longitudinal study on the relationship between circulating PCSK9 and the prediction of clinical cardiovascular outcomes in Chinese individuals with T2DM. Previous studies reported increased plasma PCSK9 levels in patients with T2DM [18, 21, 22]. Moreover, among young participants, plasma PCSK9 levels are also increased in T1DM; as glycemic control deteriorates, plasma PCSK9 levels increase significantly [23]. Insulin resistance is one of the primary causes of diabetes. Previous studies have shown that insulin resistance and subsequent hyperinsulinemia can enhance plasma circulating PCSK9 levels, and that the increase in plasma PCSK9 levels is related to poor glycemic control in patients with diabetes. Additionally, insulin resistance plays a crucial role in the homeostasis of PCSK9 in severe obesity. The PCSK9 controls the expression of LDLR on the surface of pancreatic cells, which may help maintain appropriate physiological balance to limit cholesterol overload in β cells. Pharmacological studies have shown that liraglutide can inhibit the expression of PCSK9 in HepG2 cells and db/db mice via a hepatocyte nuclear factor 1 alpha-dependent mechanism. Notably, patients with diabetes accompanied by other metabolic disorders, especially atherosclerotic cardiovascular disease, have an increased probability of experiencing cardiovascular events [24, 25]. Additionally, atherosclerotic dyslipidemia plays a vital role in the prognosis of diabetes and atherosclerotic cardiovascular diseases [26]. Even with standard treatments, patients with CHD and diabetes have an increased cardiovascular risk. Therefore, further in-depth understanding of the cardiovascular risk in CHD patients with diabetes may help to prevent and better manage such patients [4, 8, 27].

PCSK9 binds to LDLR, leading to their intracellular degradation, thereby increasing plasma LDL-C levels and hyperlipidemia [11]. Soluble PCSK9 binds to the epidermal growth factor homologous domain of LDLR, which is mainly located on the surface of liver cells, thereby preventing it from adhering to LDL-C particles [28]. In addition, it promotes LDLR degradation by enhancing endocytosis and preventing recycling [29]. In addition to the classical pathway, PCSK9 regulates lipid metabolism via the intracellular endogenous PCSK9 regulation of ApoB expression and Lp(a) metabolism [30]. PCSK9 is associated with endothelial cell apoptosis, macrophage cholesterol efflux, intracellular mitochondrial dysfunction, and inflammation, all of which lead to metabolic disorders [27, 31]. Indeed, it has recently been suggested that high levels of circulating PCSK9 could directly promote the occurrence and progression of atherosclerosis, independently of the LDL-C levels [32]. Our data from the CHD group and non-CHD group, as well as the MACEs group and non-MACEs group, also support this viewpoint. In recent years, many studies have explored the role of PCSK9 in atherosclerosis; however, the predictive role of PCSK9 in cardiovascular outcomes remains controversial [33, 34]. PCSK9 expression is positively correlated with cardiovascular events in patients with acute coronary syndrome [35]. However, in a prospective cohort study on primary prevention, the results showed that baseline plasma level of PCSK9 could not predict future cardiovascular events [36]. Therefore, further evidence is required to support PCSK9 as a new predictor of MACEs, particularly in patients with CHD and T2DM.

PCSK9 potentially interacted with several proteins, including ApoB, ApoA1, APOE, LDLR, LRP1, SORT1 and so on. Interestingly, PCSK9 was related to SORT1, which was also named Sortilin, functions as a sorting receptor in the Golgi compartment and as a clearance receptor on the cell surface [37]. It could bind and mediate degradation of lipoprotein lipase that indicate that PCSK9 may be related to SORT1 in CHD severity, and MACEs in patients with T2DM [38, 39]. These results may guide future studies of the functional interactions of PCSK9.In addition, GO term annotation showed that these proteins were mainly involved in cholesterol metabolic process, lipoprotein particle and lipoprotein particle receptor binding. A KEGG pathway analysis indicated enrichment in the cholesterol metabolism pathway. These could provide more information for us to further study the mechanism of PCSK9 in the severity of CHD and MACEs in patients with T2DM.

Previous studies have shown that PCSK9 is positively correlated with some traditional risk factors for atherosclerosis, and our study also confirmed a significant positive correlation between PCSK9 and TC and LDL-C. Previous studies have explored whether serum PCSK9 levels may be a new regulatory biomarker for the early diagnosis of atherosclerosis in T2DM. Furthermore, PCSK9 inhibitors can significantly reduce TC and LDL-C levels in patients with T2DM or atherosclerotic cardiovascular disease [40, 41]. Regardless of sex, the patients had high non-HDL-C and LDL-C levels. In the previous FOURIER and ODYSSEY trials, long-term follow-up showed that, the PCSK9 inhibitor alirocumab was more effective in reducing the occurrence of MACEs than the placebo control group [42, 43]. Currently, PCSK9 inhibitors are an effective treatment options for patients with type 2 diabetes and mixed dyslipidemia.

Several studies have shown that plasma PCSK9 levels are significantly higher in patients with CHD than in healthy individuals [20, 44, 45]. Existing epidemiological and clinical research results have shown that PCSK9 levels are high in patients with diabetes. In our study, in a group of Chinese Han T2DM patients, the plasma level of PCSK9 was positively correlated with the severity of coronary heart disease, as shown by the linear correlation results (diseased vessels: r = 0.35, p < 0.001; GS: r = 0.46, *p* < 0.001, significantly). To date, only Armentaro et al. have reported similar results in Caucasian populations [20]. It is important to emphasize how the genetic background affects clinical outcomes. The circulating plasma levels of PCSK9 vary significantly among individuals and may be influenced by genetic factors. Previous reports have shown differences in PCSK9 levels among people of different races, with Hispanics having higher levels than African–Americans and European–Americans [4]. In addition, Lakoski et al. studied the effect of PCSK9 allele sequence variation on plasma levels, and showed that African–Americans are heterozygous for PCSK9 nonsense mutations. Compared to people without invalid alleles, the median value of PCSK9 was low [44]. Through a six-year follow-up, our study not only proved that the PCSK9 level is proportional to the severity of CHD in Chinese Han T2DM patients, but also found that patients with a higher level of PCSK9 are more prone to MACEs.

## Conclusion

Our study suggests a positive correlation between PCSK9 and multi-vessel CHD, and GS and indicates that, PCSK9 is an independent risk factor for CHD. Elevated PCSK9 levels are an independent predictor of MACEs in T2DM patients and T2DM patients with CHD.

This study has some limitations. First, only two centers participated in the study, which may have led to a selection bias in the results obtained. Some conclusions should be verified in more extensive multicenter studies. Second, our study lacked healthy individuals or those with pre-diabetes; therefore, we are unsure whether PCSK9 affects diabetes. Therefore, future studies should focus on this type of cohort. However, our data provide evidence for the clinical significance of PCSK9 levels in CHD and the risk of MACEs in T2DM patients.

### Electronic supplementary material

Below is the link to the electronic supplementary material.

**Supplementary Material 1**: Figure 1. Protein–protein interaction network of PCSK9 by using STRING

**Supplementary Material 2**: Figure 2. Enrichment analysis of PCSK9 functional networks in HNSC. Significantly enriched Gene Ontology annotations and Kyoto Encyclopedia of Genes and Genomes pathways of PCSK9. CC: Cellular component; BP: Biological process; MF: Molecular function

**Supplementary Material 3**: Baseline characteristics of type 2 diabetic patients with coronary heart disease at different circulating PCSK9 levels

**Supplementary Material 4**: The relationship between PCSK9 level and the MACEs outcomes in Non-CHD group

**Supplementary Material 5**: The relationship between PCSK9 level and the MACEs outcomes in CHD patients

**Supplementary Material 6**: The relationship between PCSK9 level and the MACEs outcomes in all patients

**Supplementary Material 7**: The relationship between PCSK9 level and the MACEs outcomes in male patients

*Supplementary Material 8*: The relationship between PCSK9 level and the MACEs outcomes in female patients

**Supplementary Material 9**: Multivariate analysis of PCSK9 levels in relation to MACEs in all patients

## Data Availability

The datasets used and/or analyzed during the current study are available from the corresponding author on reasonable request.
