# Supplementary figures and images for: Association between serum PCSK9 and coronary heart disease in patients with type 2 diabetes mellitus
Source: Diabetol Metab Syndr. 2023 Dec 20;15:260. doi: 10.1186/s13098-023-01238-z (PMC10731704; doi:10.1186/s13098-023-01238-z)

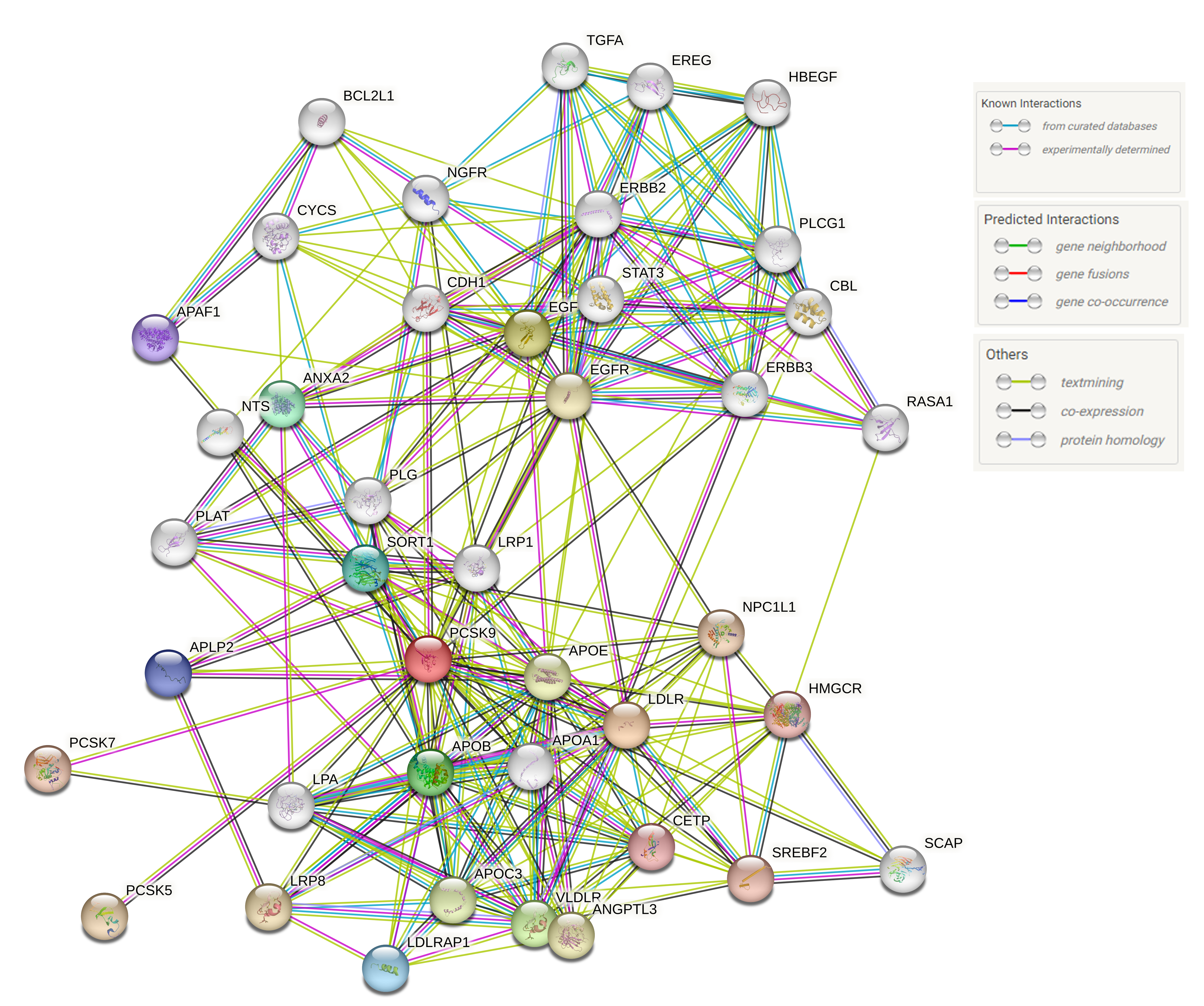

Supplement: Supplementary file 1 — Supplementary Material 1: Figure 1. Protein–protein interaction network of PCSK9 by using STRING [file 13098_2023_1238_MOESM1_ESM.png]

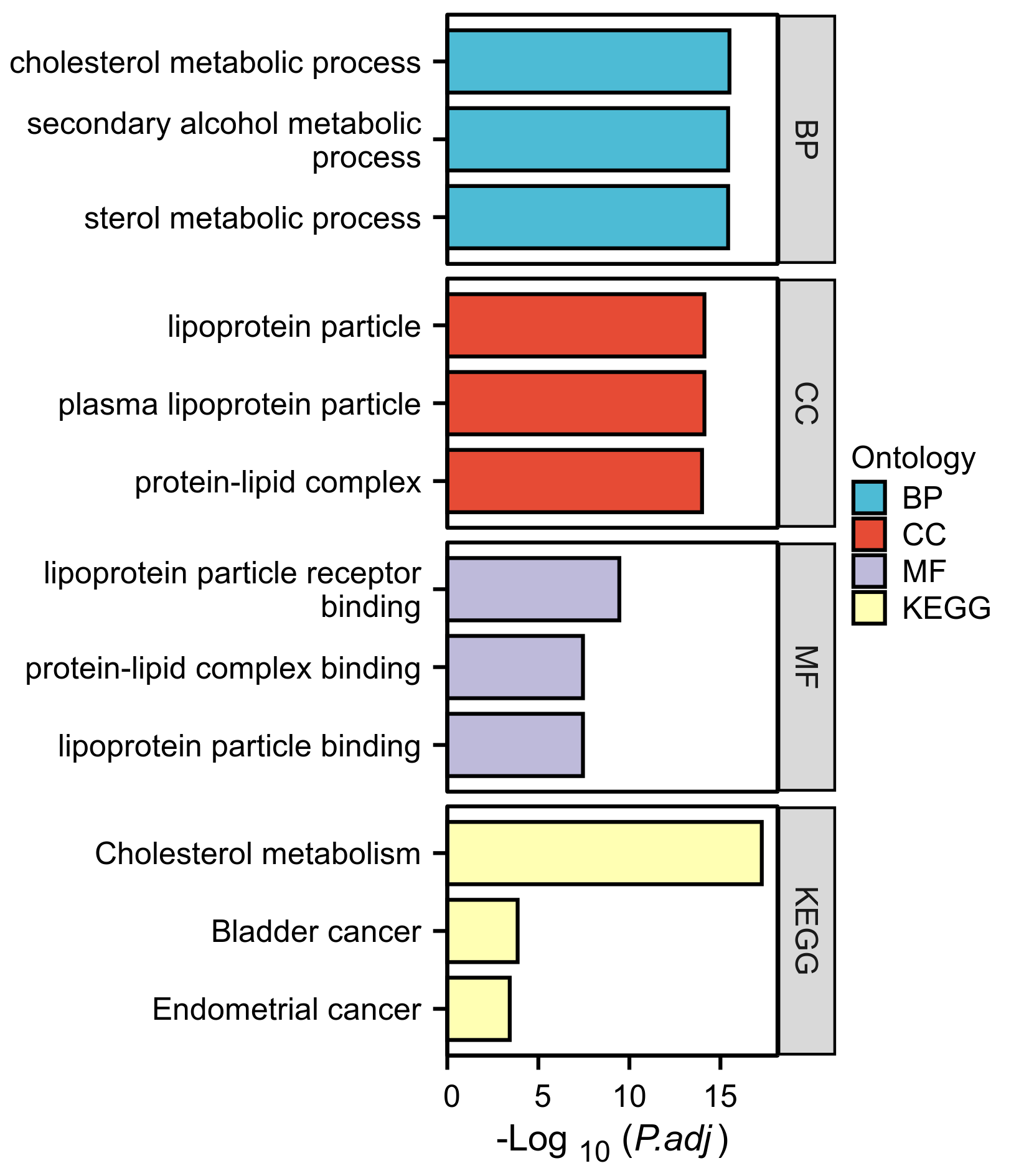

Supplement: Supplementary file 2 — Supplementary Material 2: Figure 2. Enrichment analysis of PCSK9 functional networks in HNSC. Significantly enriched Gene Ontology annotations and Kyoto Encyclopedia of Genes and Genomes pathways of PCSK9. CC: Cellular component; BP: Biological process; MF: Molecular function [file 13098_2023_1238_MOESM2_ESM.png]
