# Supplementary material for: Association between serum PCSK9 and coronary heart disease in patients with type 2 diabetes mellitus
Source: Diabetol Metab Syndr. 2023 Dec 20;15:260. doi: 10.1186/s13098-023-01238-z (PMC10731704; doi:10.1186/s13098-023-01238-z)
Supplement: Supplementary file 3 — Supplementary Material 3: Baseline characteristics of type 2 diabetic patients with coronary heart disease at different circulating PCSK9 levels [file 13098_2023_1238_MOESM3_ESM.docx]

Supplementary Table1. Baseline characteristics of type 2 diabetic patients with coronary heart disease patients at different circulating PCSK9 levels

| **Variables** | **PCSK9 concentration (ng/mL)** | | | | ***p* value** |
| --- | --- | --- | --- | --- | --- |
|  | Q1: < 430.45 | Q2: 430.45 – 554.62 | Q3: 554.62 – 695.56 | Q4: > 695.56 |  |
| Clinical characteristics |  |  |  |  |  |
| N (%) | 445 (25.06 %) | 445 (25.06%) | 444 (25.00 %) | 442 (24.88 %) | – |
| Age (years) | 56.82 ± 8.84 | 57.55 ± 9.98 | 57.23 ± 10.20 | 59.65 ± 9.78 | 0.073 |
| Male (%) | 258 (57.98 %) | 251 (56.40%) | 268 (60.36 %) | 269 (60.86 %) | 0.495 |
| BMI (kg/m^2^) | 24.83 ± 2.68 | 25.67 ± 3.19 | 25.87 ± 2.77 | 24.87 ± 2.94 | 0.214 |
| Hypertension (%) | 302 (67.87%) | 312 (70.11%) | 310 (69.82%) | 328 (74.21%) | 0.209 |
| Smoking (%) | 129(28.99%) | 116(26.07%) | 121(27.25%) | 115(26.02%) | 0.727 |
| Alcohol consumption (%) | 137(30.79%) | 130(29.21%) | 128(28.83%) | 146(33.03%) | 0.515 |
| Family history of CHD (%) | 121(26.04%) | 130(26.87%) | 144(29.57%) | 136(31.59%) | 0.365 |
| Family history of MD (%) | 164(36.85%) | 175(39.33%) | 156(35.14%) | 160(36.20%) | 0.612 |
| Laboratory variables |  |  |  |  |  |
| FPG (mmol/L) | 7.73 ± 1.11 | 7.73 ± 1.33 | 7.84 ± 1.34 | 7.64 ± 0.97 | 0.598 |
| HbA1c (%) | 7.54 ± 1.04 | 7.65 ± 1.30 | 7.64 ± 1.28 | 7.36 ± 0.88 | 0.109 |
| ApoB (mg/dL) | 81.20 (32.00) | 79.81 (32.68) | 88.82 (37.47)^b^ | 98.36 (32.74)^abc^ | <0.001 |
| ApoA1 (mg/dL) | 151.20 (28.75) | 141.61 (35.81)^a^ | 139.30 (47.28)^a^ | 136.43 (37.65)^a^ | 0.001 |
| Total cholesterol (mmol/L) | 4.36 (1.70) | 4.16 (1.54) | 4.41 (1.88)^b^ | 4.85 (1.80)^abc^ | <0.001 |
| Triglycerides (mmol/L) | 1.29 (0.72) | 1.23 (0.89) | 1.37 (0.84) | 1.35 (0.86) | 0.643 |
| HDL-C (mmol/L) | 1.17 (0.28) | 1.10 (0.28)^a^ | 1.12 (0.30)^a^ | 1.08 (0.28)^a^ | 0.001 |
| LDL-C (mmol/L) | 2.59 (1.22) | 2.51 (1.20) | 2.85 (1.48)^ab^ | 3.19 (1.29)^ab^ | <0.001 |
| Lp(a) (nmol/L) | 27.43 (15.27) | 38.55 (22.51)^a^ | 49.19 (45.27)^ab^ | 99.70 (99.48)^abc^ | <0.001 |
| hs-CRP (mg/L) | 1.44 (1.67) | 1.80 (1.89) | 1.76 (1.88) | 1.37 (1.66) | 0.946 |
| HCY (umol/L) | 11.19 (7.37) | 11.43 (7.59) | 11.60 (7.81) | 14.38 (11.53)^abc^ | <0.001 |
| sdLDL-C (mmol/L) | 0.75 (0.52) | 0.68 (0.49) | 0.78 (0.45) | 0.81 ( 0.47)^ab^ | 0.022 |
| PCSK9 (ng/mL) | 360.12 (80.90) | 498.32 (71.41) | 604.77 (68.38) | 800.72 (129.80) |  |
| Diseased vessels in CHD patients |  |  |  |  |  |
| One vessel (%) | 192 (43.14%) | 176 (39.55%) | 106 (23.87%)^ab^ | 55 (12.44%)^abc^ | <0.001 |
| Two vessels (%) | 176 (39.55%) | 135 (30.34%)^a^ | 114 (25.68%)^a^ | 93 (21.04%)^ab^ | <0.001 |
| Three vessels (%) | 77 (17.30%) | 138 (31.01%)^a^ | 224 (50.45%)^ab^ | 294 (66.52%)^abc^ | <0.001 |
| Gensini score | 23 (21) | 30 (21)^a^ | 36 (24)^ab^ | 46 (20)^abc^ | <0.001 |

Data are reported as means ± SD or n(%), median (interquartile ranges). SD: Standard deviation

BMI: body mass index; FPG: fasting plasma glucose; HbA1c: Hemoglobin A1c; apoB: apolipoprotein B; apoA1: apolipoprotein A1; HDL-C: high density lipoprotein cholesterol; LDL-C: low density lipoprotein cholesterol; Lp(a): lipoprotein (a); Hs-CRP: hypersensitive C-reactive protein; HCY: homocysteine; sdLDL-C: small dense low-density lipoprotein cholesterol; PCSK9: proprotein convertase enzyme subtilisin/kexin type 9.

Statistical analysis was performed with the ANOVA or Kruskal – Wall test and with Chi-square test for categorical variables.

a: Shows that the *p* < 0.05 compared with the Q1 group.

b: Shows that the *p* < 0.05 compared with the Q2 group.

c: Shows that the *p* < 0.05 compared with the Q3 group.
