# Supplementary material for: Association between serum PCSK9 and coronary heart disease in patients with type 2 diabetes mellitus
Source: Diabetol Metab Syndr. 2023 Dec 20;15:260. doi: 10.1186/s13098-023-01238-z (PMC10731704; doi:10.1186/s13098-023-01238-z)
Supplement: Supplementary file 4 — Supplementary Material 4: The relationship between PCSK9 level and the MACEs outcomes in Non-CHD group [file 13098_2023_1238_MOESM4_ESM.docx]

Supplementary Table 2. The relationship between PCSK9 level and the MACEs outcomes in Non-CHD group

| **MACEs** | **PCSK9 concentration (ng/mL)** | | | | ***p*** |
| --- | --- | --- | --- | --- | --- |
|  | Q1: < 432.98 | Q2: 432.98 – 521.98 | Q3: 521.98 – 621.24 | Q4: > 621.24 |  |
|  | n = 310 | n = 456 | n = 336 | n = 106 |  |
| cardiovascular deaths | 0 (0.00%) | 2 (0.44%) | 1 (0.30%) | 1 (0.94%) | 0.439 |
| non-fatal MI | 4 (1.29%) | 10 (2.19%) | 9 (2.68%) | 5 (4.72%) | 0.155 |
| non-fatal strokes | 2 (0.65%) | 8 (1.75%) | 8 (2.38%) | 4 (3.77%) | 0.109 |
| heart failure | 1 (0.32%) | 7 (1.54%) | 6 (1.79%) | 3 (2.83%) | 0.157 |
| hospitalization for unstable angina | 2 (0.65%) | 10 (2.19%) | 8 (2.38%) | 5 (4.72%)^a^ | 0.047 |
| total | 9 (2.90%) | 37 (8.11%)^a^ | 32 (9.52%)^a^ | 18 (16.98%)^abc^ | < 0.001 |

PCSK9: Proprotein convertase subtilisin/kexin type 9. CHD: Coronary heart disease. MACEs: major cardiovascular events.

Statistical analysis was performed with Chi-square test for categorical variables.

a: Shows that the *p* < 0.05 compared with the Q1 group.

b: Shows that the *p* < 0.05 compared with the Q2 group.

c: Shows that the *p* < 0.05 compared with the Q3 group.
