# Supplementary material for: Association between serum PCSK9 and coronary heart disease in patients with type 2 diabetes mellitus
Source: Diabetol Metab Syndr. 2023 Dec 20;15:260. doi: 10.1186/s13098-023-01238-z (PMC10731704; doi:10.1186/s13098-023-01238-z)
Supplement: Supplementary file 5 — Supplementary Material 5: The relationship between PCSK9 level and the MACEs outcomes in CHD patients [file 13098_2023_1238_MOESM5_ESM.docx]

Supplementary Table 3. The relationship between PCSK9 level and the MACEs outcomes in CHD patients

| **MACEs** | **PCSK9 concentration (ng/mL)** | | | | ***p*** |
| --- | --- | --- | --- | --- | --- |
|  | Q1: < 432.98 | Q2: 432.98 – 521.98 | Q3: 521.98 –621.24 | Q4: > 621.24 |  |
|  | n = 438 | n = 288 | n = 410 | n = 640 |  |
| cardiovascular deaths | 4 (0.91%) | 4 (1.39%) | 8 (1.95%) | 13 (2.03%) | 0.367 |
| non-fatal MI | 14 (3.20%) | 12 (4.17%) | 19 (4.63%) | 57 (8.91%)^abc^ | < 0.001 |
| non-fatal strokes | 9 (2.05%) | 8 (2.78%) | 12 (2.93%) | 23 (3.59%) | 0.344 |
| heart failure | 7 (1.60%) | 6 (2.08%) | 10 (2.44%) | 19 (2.97%) | 0.353 |
| hospitalization for unstable angina | 7 (1.60%) | 9 (3.13%) | 12 (2.93%) | 24 (3.75%) | 0.132 |
| total | 41 (9.36%) | 39 (13.54%) | 61 (14.88%)^a^ | 136 (21.25%)^abc^ | < 0.001 |

PCSK9: Proprotein convertase subtilisin/kexin type 9. CHD: Coronary heart disease. MACEs: major cardiovascular events.

Statistical analysis was performed with Chi-square test for categorical variables.

a: Shows that the *p* < 0.05 compared with the Q1 group.

b: Shows that the *p* < 0.05 compared with the Q2 group.

c: Shows that the *p* < 0.05 compared with the Q3 group.
