# Supplementary material for: Association between serum PCSK9 and coronary heart disease in patients with type 2 diabetes mellitus
Source: Diabetol Metab Syndr. 2023 Dec 20;15:260. doi: 10.1186/s13098-023-01238-z (PMC10731704; doi:10.1186/s13098-023-01238-z)
Supplement: Supplementary file 6 — Supplementary Material 6: The relationship between PCSK9 level and the MACEs outcomes in all patients [file 13098_2023_1238_MOESM6_ESM.docx]

Supplementary Table 4. The relationship between PCSK9 level and the MACEs outcomes in all patients

| MACEs | PCSK9 concentration (ng/mL) | | | | *p* |
| --- | --- | --- | --- | --- | --- |
|  | Q1: < 432.98 | Q2: 432.98 – 521.98 | Q3: 521.98 –621.24 | Q4: > 621.24 |  |
|  | n = 748 | n = 744 | n = 746 | n = 746 |  |
| cardiovascular deaths | 4 (0.53%) | 6 (0.81%) | 9 (1.21%) | 14 (1.88%)^ab^ | 0.030 |
| non-fatal MI | 18 (2.41%) | 22 (2.96%) | 28 (3.75%) | 62 (8.31%)^abc^ | < 0.001 |
| non-fatal strokes | 11 (1.47%) | 16 (2.15%) | 20 (2.68%) | 27 (3.62%)^ab^ | 0.016 |
| heart failure | 8 (1.07%) | 13 (1.75%) | 16 (2.14%) | 22 (2.95%)^a^ | 0.023 |
| hospitalization for unstable angina | 9 (1.20%) | 19 (2.55%)^a^ | 20 (2.68%)^a^ | 29 (3.89%)^a^ | 0.003 |
| total | 50 (6.68%) | 76 (10.22%)^a^ | 93 (12.47%)^a^ | 154 (20.64%)^abc^ | < 0.001 |

PCSK9: Proprotein convertase subtilisin/kexin type 9. CHD: Coronary heart disease. MACEs: major cardiovascular events.

Statistical analysis was performed with Chi-square test for categorical variables.

a: Shows that the *p* < 0.05 compared with the Q1 group.

b: Shows that the p < 0.05 compared with the Q2 group.

c: Shows that the *p* < 0.05 compared with the Q3 group.
