# Supplementary material for: Association between serum PCSK9 and coronary heart disease in patients with type 2 diabetes mellitus
Source: Diabetol Metab Syndr. 2023 Dec 20;15:260. doi: 10.1186/s13098-023-01238-z (PMC10731704; doi:10.1186/s13098-023-01238-z)
Supplement: Supplementary file 7 — Supplementary Material 7: The relationship between PCSK9 level and the MACEs outcomes in male patients [file 13098_2023_1238_MOESM7_ESM.docx]

Supplementary Table 5. The relationship between PCSK9 level and the MACEs outcomes in male patients

| MACEs | PCSK9 concentration (ng/mL) | | | | *p* |
| --- | --- | --- | --- | --- | --- |
|  | Q1: < 432.98 | Q2: 432.98 – 521.98 | Q3: 521.98 –621.24 | Q4: > 621.24 |  |
|  | n = 429 | n = 406 | n = 457 | n = 440 |  |
| cardiovascular deaths | 3 (0.70%) | 3 (0.74%) | 6 (1.31%) | 7 (1.59%) | 0.388 |
| non-fatal MI | 11 (2.56%) | 14 (3.45%) | 18 (3.94%) | 38 (8.64%)^abc^ | < 0.001 |
| non-fatal strokes | 6 (1.40%) | 10 (2.46%) | 13 (2.84%) | 16 (3.64%) | 0.116 |
| heart failure | 5 (1.17%) | 8 (1.97%) | 10 (2.19%) | 13 (2.95%) | 0.194 |
| hospitalization for unstable angina | 4 (0.93%) | 12 (2.96%)^a^ | 12 (2.63%)^a^ | 17 (3.86%)^a^ | 0.025 |
| total | 29 (6.76%) | 47 (11.58%)^a^ | 59 (12.91%)^a^ | 91 (20.68%)^abc^ | < 0.001 |

PCSK9: Proprotein convertase subtilisin/kexin type 9. CHD: Coronary heart disease. MACEs: major cardiovascular events.

Statistical analysis was performed with Chi-square test for categorical variables.

a: Shows that the *p* < 0.05 compared with the Q1 group.

b: Shows that the p < 0.05 compared with the Q2 group.

c: Shows that the *p* < 0.05 compared with the Q3 group.
