# Supplementary material for: Association between serum PCSK9 and coronary heart disease in patients with type 2 diabetes mellitus
Source: Diabetol Metab Syndr. 2023 Dec 20;15:260. doi: 10.1186/s13098-023-01238-z (PMC10731704; doi:10.1186/s13098-023-01238-z)
Supplement: Supplementary file 8 — Supplementary Material 8: The relationship between PCSK9 level and the MACEs outcomes in female patients [file 13098_2023_1238_MOESM8_ESM.docx]

Supplementary Table 6. The relationship between PCSK9 level and the MACEs outcomes in female patients

| MACEs | PCSK9 concentration (ng/mL) | | | | *p* |
| --- | --- | --- | --- | --- | --- |
|  | Q1: < 432.98 | Q2: 432.98 – 521.98 | Q3: 521.98 –621.24 | Q4: > 621.24 |  |
|  | n = 319 | n = 338 | n = 289 | n = 306 |  |
| cardiovascular deaths | 1 (0.31%) | 3 (0.89%) | 3 (1.04%) | 7 (2.29%) | 0.068 |
| non-fatal MI | 7 (2.19%) | 8 (2.37%) | 10 (3.46%) | 24 (7.84%)^abc^ | < 0.001 |
| non-fatal strokes | 5 (1.57%) | 6 (1.78%) | 7 (2.42%) | 11 (3.59%) | 0.190 |
| heart failure | 3 (0.94%) | 5 (1.48%) | 6 (2.08%) | 9 (2.94%) | 0.172 |
| hospitalization for unstable angina | 5 (1.58%) | 7 (2.07%) | 8 (2.77%) | 12 (3.92%) | 0.151 |
| total | 21 (6.58%) | 29 (8.58%) | 34 (11.76%)^a^ | 63 (20.59%)^abc^ | < 0.001 |

PCSK9: Proprotein convertase subtilisin/kexin type 9. CHD: Coronary heart disease. MACEs: major cardiovascular events.

Statistical analysis was performed with Chi-square test for categorical variables.

a: Shows that the *p* < 0.05 compared with the Q1 group.

b: Shows that the p < 0.05 compared with the Q2 group.

c: Shows that the *p* < 0.05 compared with the Q3 group.
