# Supplementary material for: Association between serum PCSK9 and coronary heart disease in patients with type 2 diabetes mellitus
Source: Diabetol Metab Syndr. 2023 Dec 20;15:260. doi: 10.1186/s13098-023-01238-z (PMC10731704; doi:10.1186/s13098-023-01238-z)
Supplement: Supplementary file 9 — Supplementary Material 9: Multivariate analysis of PCSK9 levels in relation to MACEs in all patients [file 13098_2023_1238_MOESM9_ESM.docx]

Supplementary table7. Multivariate analysis of PCSK9 levels in relation to MACEs in all patients

|  | **b value** | **Hazard Ratio** | **95% Confidence Interval** | ***p* value** |
| --- | --- | --- | --- | --- |
| Clinical characteristics |  |  |  |  |
| Age (years) | 0.006 | 1.006 | 0.996 – 1.016 | 0.268 |
| Male (%) | 0.104 | 1.110 | 0.894 – 1.378 | 0.346 |
| BMI (kg/m^2^) | - 0.001 | 0.999 | 0.973 – 1.025 | 0.921 |
| Hypertension (%) | 0.246 | 1.279 | 1.011 – 1.617 | 0.040 |
| Smoking (%) | - 0.048 | 0.953 | 0.753 – 1.205 | 0.688 |
| Alcohol consumption (%) | - 0.044 | 0.957 | 0.752 – 1.218 | 0.721 |
| Family history of CHD (%) | 0.306 | 1.358 | 1.074 – 1.716 | 0.011 |
| Family history of MD (%) | 0.075 | 1.078 | 0.873 – 1.330 | 0.486 |
| Laboratory variables |  |  |  |  |
| FBG (mmol/L) | - 0.009 | 0.991 | 0.767 – 1.281 | 0.947 |
| HbA1c (%) | 0.006 | 1.006 | 0.998 – 1.014 | 0.135 |
| ApoB (mg/dL) | 0.007 | 1.007 | 0.999 – 1.014 | 0.093 |
| ApoA1 (mg/dL) | 0.002 | 1.002 | 0.998 – 1.007 | 0.238 |
| Total cholesterol (mmol/L) | - 0.044 | 0.957 | 0.778 – 1.179 | 0.681 |
| Triglycerides (mmol/L) | - 0.032 | 0.968 | 0.839 – 1.117 | 0.657 |
| HDL-C (mmol/L) | - 0.076 | 0.927 | 0.612 – 1.405 | 0.722 |
| LDL-C (mmol/L) | 0.026 | 1.026 | 0.797 – 1.322 | 0.840 |
| Lp(a) (nmol/L) | 0.000 | 1.000 | 0.997 – 1.002 | 0.816 |
| hs-CRP (mg/L) | - 0.058 | 0.944 | 0.873 – 1.020 | 0.143 |
| HCY (umol/L) | - 0.019 | 0.981 | 0.964 – 0.998 | 0.031 |
| sdLDL-C (mmol/L) | 0.380 | 1.463 | 1.062 – 2.016 | 0.020 |
| Quartile of PCSK9 (ng/mL) |  |  |  |  |
| Q1 | – | – | – | – |
| Q2 | 0.435 | 1.505 | 1.053 – 2.151 | 0.025 |
| Q3 | 0.502 | 1.625 | 1.142 – 2.313 | 0.007 |
| Q4 | 1.030 | 2.780 | 1.930 – 4.004 | < 0.001 |

BMI: body mass index; FPG: fasting plasma glucose; HbA1c: Hemoglobin A1c; apoB: apolipoprotein B; apoA1: apolipoprotein A1; HDL-C: high density lipoprotein cholesterol; LDL-C: low density lipoprotein cholesterol; Lp(a): lipoprotein (a); Hs-CRP: hypersensitive C-reactive protein; HCY: homocysteine; sdLDL-C: small dense low-density lipoprotein cholesterol; PCSK9: proprotein convertase enzyme subtilisin/kexin type 9.
